# Perturbation of nuclear–cytosolic shuttling of Rx1 compromises extreme resistance and translational arrest of potato virus X transcripts

**DOI:** 10.1111/tpj.15179

**Published:** 2021-03-23

**Authors:** Manon M. S. Richard, Marijn Knip, Joëlle Schachtschabel, Machiel S. Beijaert, Frank L. W. Takken

**Affiliations:** ^1^ Molecular Plant Pathology Swammerdam Institute for Life Sciences (SILS) University of Amsterdam Amsterdam the Netherlands

**Keywords:** NLR, virus, plant immunity, cell death, translational inhibition, *Nicotiana benthamiana*

## Abstract

Many plant intracellular immune receptors mount a hypersensitive response (HR) upon pathogen perception. The concomitant localized cell death is proposed to trap pathogens, such as viruses, inside infected cells, thereby preventing their spread. Notably, extreme resistance (ER) conferred by the potato immune receptor Rx1 to potato virus X (PVX) does not involve the death of infected cells. It is unknown what defines ER and how it differs from HR‐based resistance. Interestingly, Rx1 can trigger an HR, but only upon artificial (over)expression of PVX or its avirulence coat protein (CP). Rx1 has a nucleocytoplasmic distribution and both pools are required for HR upon transient expression of a PVX‐GFP amplicon. It is unknown whether mislocalized Rx1 variants can induce ER upon natural PVX infection. Here, we generated transgenic *Nicotiana benthamiana* producing nuclear‐ or cytosol‐restricted Rx1 variants. We found that these variants can still mount an HR. However, nuclear‐ or cytosol‐restricted Rx1 variants can no longer trigger ER or restricts viral infection. Interestingly, unlike the mislocalized Rx1 variants, wild‐type Rx1 was found to compromise CP protein accumulation. We show that the lack of CP accumulation does not result from its degradation but is likely to be linked with translational arrest of its mRNA. Together, our findings suggest that translational arrest of viral genes is a major component of ER and, unlike the HR, is required for resistance to PVX.

## Significance Statement

It is unknown how antiviral immunity is conferred in the case of extreme resistance. We show that extreme resistance correlates with a translational arrest of viral transcripts and can be uncoupled from the hypersensitive response, which was observed to not be required for viral immunity.

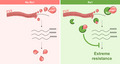

## INTRODUCTION

The mechanisms underlying *R*‐gene‐mediated antiviral immunity in plants are largely unknown. As obligate intracellular parasites, viruses rely on the translational machinery of the host for their protein synthesis. In fact, many viruses are known to manipulate and/or boost the translation capacity of the host cell to allow viral replication (Gale *et al.,* 2000; Jaafar and Kieft, 2019; Wu *et al.,* 2019). Preventing abuse of the translational machinery by viruses therefore constitutes an excellent defence strategy. In recent studies, plant immunity has been linked to translation control, although the underlying mechanism and its importance in resistance against viruses is not fully understood (Bhattacharjee *et al.,* 2009; Meteignier *et al.,* 2017; Zorzatto *et al.,* 2015). Here, we show that the plant immune receptor Rx1 prevents the translation of potato virus X (PVX)–coat protein (CP) transcripts.

Plants have evolved different layers of defence to counteract viral infection. Viruses hijack the host cellular machinery for their own benefit. As viruses lack protein translation machinery, they have to compete with host mRNAs to recruit ribosomes and produce proteins (Au and Jan, 2014; Walsh *et al.,* 2013; Wang, 2015). Several recessive genes conferring viral resistance correspond to mutations in genes involved in translation, making these mutant proteins incompatible for viral manipulation (Robaglia and Caranta, 2006; Sanfaçon, 2015). Additionally, among eukaryotes an effective RNA silencing machinery is paramount in the defence against viruses. Infection with any type of virus generates double‐stranded viral RNAs (dsRNAs) (Li and Wang, 2019). dsRNAs are recognized and processed by dsRNA‐specific RNAses, called Dicer‐like (DCL) proteins, into viral small interfering RNA (vsiRNA). These vsiRNAs are subsequently incorporated into an Argonaute (AGO) protein and loaded onto an RNA‐induced silencing complex (RISC). In this RISC complex the vsiRNAs act as guide molecules to target homologous viral RNA for degradation or translational repression (Li and Wang, 2019). To counteract this response, viruses carry silencing suppressors. These viral suppressors of RNA silencing (VSRs) target different pathways and/or components of the silencing machinery to compromise the plant antiviral RNA silencing mechanism to promote viral infection.

As a second layer of defence, plants have evolved intracellular immune receptors that recognize viruses, as well as other pathogens, in order to defend themselves. These resistance (R) proteins, which are often nucleotide‐binding leucine‐rich repeat receptors (NLRs), mount an immune response upon recognition of a specific pathogen‐derived avirulence factor (Avr). Based on their N‐terminal domain, NLRs can be divided into two major subgroups: those carrying a Toll/interleukin‐1 receptor (TIR) domain and those carrying a coiled‐coil (CC) domain, the TNLs and the CNLs, respectively. Upon the perception of Avr, both TNLs and CNLs form multimeric complex assemblies that trigger downstream signalling. Recently, structural studies have shown that the activation of the Arabidopsis CNL ZAR1 (HOPZ‐activated resistance 1) and the *Nicotiana benthamiana* ROQ1 (recognition of XopQ 1) or the Arabidopsis RPP1 (Recognition of Peronospora parasitica 1) TNLs result in oligomerization and the formation of, respectively, a pentameric or tetrameric complex assemblies called resistosomes (Ma *et al.,* 2020; Martin *et al.,* 2020; Wang *et al.,* 2019a, 2019b,2019a, 2019b). The formation of TIR–NLR resistosomes results in the exposure of the active site of a nicotinamide adenine dinucleoside (NAD) hydrolase in the TIR domain, the initiation of NAD hydrolysis and the activation of immune responses (Ma *et al.,* 2020; Martin *et al.,* 2020). The mechanism for how CNL resistosomes trigger immunity is unclear, but for ZAR1 a funnel‐shaped structure is formed that is proposed to disturb membrane integrity (Wang *et al.,* 2019a). Immune responses triggered by R proteins are manifold, but it is unknown how they halt viral multiplication and spread. A common immune output is the induction of cell death, known as the hypersensitive response (HR) (Balint‐Kurti, 2019), which is proposed to restrict pathogen progression at the site of infection. The HR has been observed in several incompatible plant–virus interactions, such as in tobacco mosaic virus (TMV)‐infected tobacco plants carrying the resistance gene *N* (Whitham *et al.,* 1994). Although NLRs typically localize at the site of action of their recognized Avr determinants, the subcellular localization from where NLR resistosomes activate immunity is still under debate (Zhang *et al.,* 2017). Whereas some NLRs are tethered to the plasma membrane, such as Arabidopsis resistance to *Pseudomonas syringae* pv. *maculicola* *1* (*RPM1*), others localize to the endomembrane system or the cytosol (Padmanabhan and Dinesh‐Kumar, 2014). Of note, for a subset of NLRs a dynamic nucleocytoplasmic distribution has been reported, such as N from tobacco (Burch‐Smith *et al.,* 2007), MLA10 from *Hordeum vulgare* (barley) (Bai *et al.,* 2012), SNC1 from Arabidopsis (Cheng *et al.,* 2009; Xu *et al.,* 2014) and Rx1 from *Solanum tuberosum* (potato) (Slootweg *et al.,* 2010; Tameling *et al.,* 2010). In these cases, disturbing the subcellular location or the ability of the protein to translocate compromises R protein function. For example, SNC1‐mediated immune responses (cell death induction) were increased when this protein was artificially targeted to the nucleus (Cheng *et al.,* 2009). Enforcing a cytosolic location for the barley CNL MLA10 enhanced its capacity to trigger cell death, whereas targeting MLA10 to the nucleus decreased the potential to induce cell death but did not compromise its ability to mount resistance against the powdery mildew fungus (*Blumeria graminis*) (Bai *et al.,* 2012). Distinct subcellular defence branches have been reported for the Arabidopsis TNL Resistance to *Pseudomonas syringae* 4 (RPS4). A nuclear localization of RPS4 is essential for bacterial growth restriction of *P. syringae* carrying AvrRps4, whereas a nucleocytoplasmic distribution of RPS4 is required for the induction of cell death (Heidrich *et al.,* 2011).

The potato CNL Rx1 confers resistance against PVX upon recognition of its CP (Bendahmane *et al.,* 1995; Goulden *et al.,* 1993). Notably, an *Rx1*‐breaking PVX strain produces a CP variant (CP^RB^) that is not recognized by Rx1 and does not trigger immune responses (Bendahmane *et al.,* 1999; Goulden *et al.,* 1993). Rx1 confers a so‐called ‘extreme resistance’ (ER) response that prevents viral replication at the single‐cell level without triggering the HR (Adams *et al.,* 1986; Kohm *et al.,* 1993). Hence, cell death is not induced by the inoculation of PVX strains avirulent on *Rx1*‐containing potato, or on transgenic *N. benthamiana* plants expressing *Rx1* (Bendahmane *et al.,* 1999). Rx1 can, nonetheless, trigger cell death when the CP from an avirulent PVX strain (CP^AVR^) or PVX infectious clone is overexpressed in plants expressing *Rx1* (transiently or stably) (Bendahmane *et al.,* 1999). The biological relevance of cell death for Rx1 immunity, and its role in resistance against PVX, is unknown. Furthermore, the mechanism by which Rx1‐mediated extreme resistance prevents PVX replication is unknown.

Like several other NLRs, Rx1 has a nucleocytoplasmic distribution (Slootweg *et al.,* 2010; Tameling *et al.,* 2010). PVX recognition is proposed to occur in the cytosol as Rx1 fails to recognize the CP when it is targeted to the nucleus (Slootweg *et al.,* 2010). Notably, the fusion of Rx1 with either a nuclear localization signal (NLS) or a nuclear exclusion signal (NES) revealed that both the nuclear and cytoplasmic Rx1 pools are important to limit viral infection and cell death induction upon transient (over)expression of a PVX‐GFP amplicon (Knip *et al.,* 2019; Slootweg *et al.,* 2010). The capacity of these mislocalized Rx1 variants to trigger (extreme) resistance against PVX upon natural infection (e.g. by using rub‐inoculation of infectious PVX‐GFP particles) has not been evaluated, and it is not known whether they can halt the virus.

Here we set out to assess whether Rx1 localization variants confer extreme resistance against PVX upon rub‐inoculation. We generated stable transgenic *N. benthamiana* lines producting Rx1 variants that are either nuclear localized or nuclear excluded. We observed that mislocalized Rx1 variants could still induce cell death but failed in containing viral spread and in mounting full resistance. Notably, unlike wild‐type Rx1, both Rx1 variants were unable to prevent CP protein accumulation in *N. benthamiana*. Our results imply that Rx1 triggers a translational arrest of PVX‐CP transcripts. Therefore, the ability of Rx1 to induce translational arrest coincides with its ability to trigger resistance against PVX without triggering cell death. Altogether, our data suggest the translational repression of viral transcripts as a major component of Rx1‐medidated antiviral ER.

## RESULTS

### Targeting Rx1 to either the cytosol or the nucleus impairs extreme resistance against PVX

**Figure 1:**
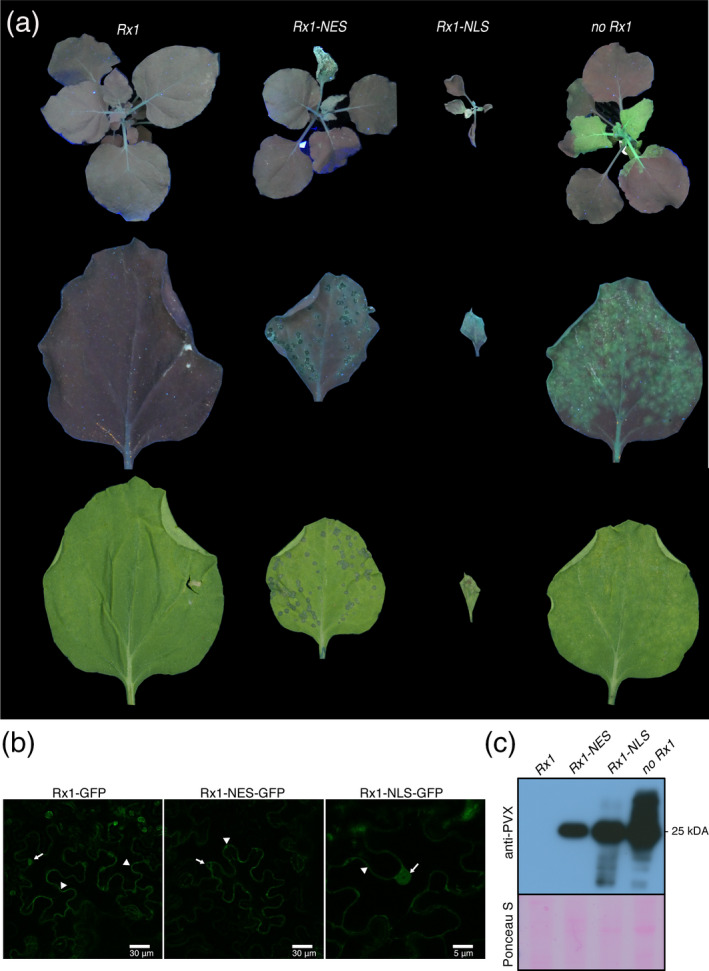
Rx1 localization variants Rx1‐NLS and Rx1‐NES failed to block PVX‐GFP infection. (a) PVX rub‐inoculated 5‐week‐old *Nicotiana benthamiana* plants at 8 days post‐inoculation (8 dpi) under UV light, and a PVX‐GFP rub‐inoculated leaf under UV and white light at 6 dpi, presented in top, middle and bottom rows, respectively. (b) Whereas Rx1 shows a nuclear–cytosolic distribution, the variants are either nuclear excluded (Rx1‐NES) or nuclear enriched (Rx1‐NLS). Subcellular localization of GFP‐tagged Rx1 and Rx1 variants visualized by confocal microscopy. The *35S_LS_::Rx1‐GFP*, *35S_LS_::Rx1‐NLS‐GFP* and *35S_LS_::Rx1‐NES‐GFP* constructs where transiently expressed in *N. benthamiana*. Arrows and arrowheads indicate the nucleus and the cytoplasm, respectively. (c) Immunodetection of PVX‐GFP in systemic leaves of rub‐inoculated *N. benthamiana* transgenic lines expressing *Rx1*, *Rx1‐NES* and *Rx1‐NLS*, 8 days after rub‐inoculation. The immunoblotting was performed using polyclonal anti‐PVX antibody followed by incubation with horseradish peroxidase (HRP)‐conjugated goat anti‐rabbit immunoglobulin G (IgG) secondary antibody. Ponceau S staining shows the equal protein loading of the samples.

The cytosolic/nuclear distribution pattern of Rx1 has been reported to modulate its immune function in *N. benthamiana* leaves in transient expression assays (Knip *et al.,* 2019; Slootweg *et al.,* 2010). Importantly, these studies did not elucidate whether the cytosolic or nuclear pool of Rx1 confers extreme resistance in response to natural viral infection. To assess the ability of nucleus‐ or cytosol‐restricted variants of Rx1 to contain the viral spread of PVX, transgenic *N. benthamiana* lines were generated that express *Rx1‐NLS* or *Rx1‐NES*. Notably, only a single fertile homozygous line could be obtained for *Rx1‐NLS*. The progeny of this line was stunted as compared with those without *Rx1* (WT) or carrying *Rx1* or *Rx1‐NES* (Figure 1a). The phenotype triggered by Rx1‐NLS resembles an autoimmune phenotype. The phenotype could not be attributed to the overexpression of *Rx1‐NLS*, as reverse transcription quantitative polymerase chain reaction (RT‐qPCR) showed that its expression level was lower than that of *Rx1‐NES*, which did not exert an apparent phenotype (Figure S1). The subcellular localization of the different Rx1 variants was assessed by confocal microscopy (Figure 1b). As previously reported, Rx1 shows a nucleocytoplasmic distribution (Knip *et al.,* 2019; Slootweg *et al.,* 2010), whereas Rx1‐NES is mainly observed in the cytosol and Rx1‐NLS is enriched in the nucleus, confirming the functionality of these constructs (Figure 1b).

Next, we assessed the capacity of the nuclear and cytosolic pool of Rx1 to confer extreme resistance against PVX. Wild type (WT, i.e. no *Rx1*), *Rx1*, *Rx1‐NLS* and *Rx1‐NES N. benthamiana* plants were rub‐inoculated with sap containing PVX‐GFP virus. The PVX‐GFP virus expresses *GFP* from a duplicated *CP* promoter (Jones *et al.,* 1999; Richard *et al.,* 2020). This recombinant strain allowed us to monitor, in a semi‐real‐time fashion, viral replication and systemic spread by visualizing GFP fluorescence using UV light. The WT *N. benthamiana* plants (without *Rx1*) inoculated with PVX‐GFP developed clear viral symptoms, such as a mosaic pattern in the tips of the youngest leaves. In addition, compared with *Rx1* plants, the WT plant became slightly stunted following PVX‐GFP inoculation, demonstrating successful infection. Under UV light, bright‐green fluorescence was apparent in foci in the inoculated leaves, whereas a more confluent GFP signal was visible in systemically infected leaves (Figure 1a), confirming that this plant species is susceptible to the virus (Figure 1a). The accumulation of viral proteins was confirmed by immunoblotting using a polyclonal antibody raised against PVX (Figure 1c). As expected, GFP fluorescence and mosaic symptoms could not be observed, neither locally nor systemically, in transgenic *Rx1* plants at 8 days post‐inoculation (8 dpi) (Figure 1a). Importantly, these plants did not show any apparent phenotype, like local lesions on the inoculated leaf, in line with the extreme resistance conferred by Rx1 (Figure 1a). The absence of the virus in systemic tissues could be confirmed with immunoblotting (Figure 1c). Together, these data confirm that, as in potato, Rx1 confers extreme resistance in *N. benthamiana* by containing the virus without an apparent HR.

In contrast, inoculated leaves of *Rx1‐NES* and *Rx1‐NLS* plants develop circular necrotic lesions at 3 dpi (Figure S2). These lesions continued to expand, eventually merging into large necrotic sectors and trailing necrosis throughout the plant (Figure S2). Under UV light, a GFP signal was observed in the inoculated and systemic leaves of both *Rx1‐NES* and *Rx1‐NLS* plants, indicating the spread of the virus. The presence of high viral titres in systemic leaves of *Rx1‐NLS* and *Rx1‐NES* plants was confirmed by immunoblotting (Figure 1c). Trailing necrosis as a result of the spread of PVX‐GFP through the upper plant parts was also visible in the *Rx1‐NES* plants at 10 dpi (Figure S2). These results show that nuclear‐ or cytosolic‐localized Rx1 variants do not provide extreme resistance, but rather a compromised resistance response resulting in systemic viral spread and trailing necrosis. Notably, Rx1‐NLS appears to trigger an autoimmune response resulting in stunted and/or non‐viable plants, but this response is insufficient to contain the virus.

### Wild‐type Rx1, but not mislocalized NLS and NES variants, block CP106^AVR^ protein accumulation

**Figure 2:**
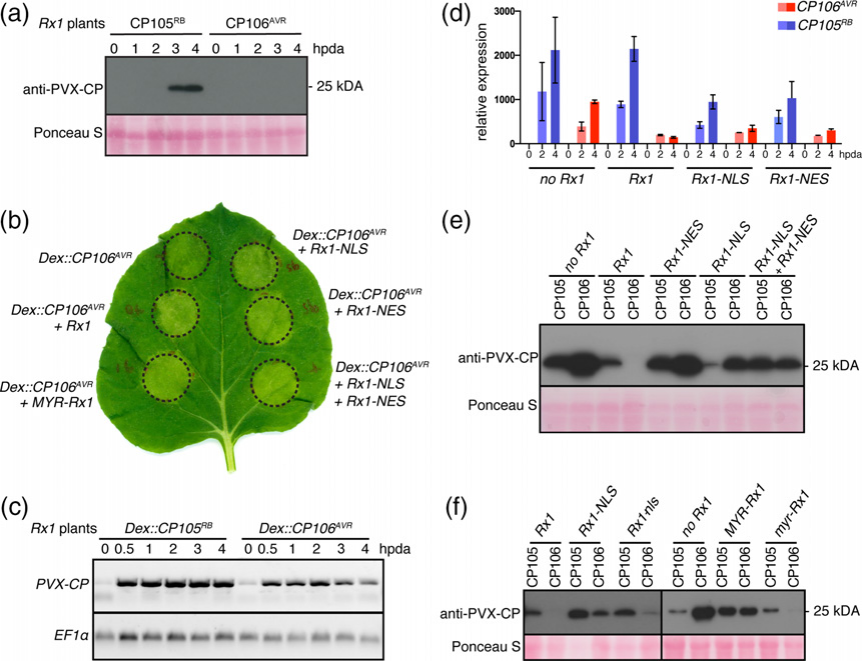
Rx1, Rx1‐NES, Rx1‐NLS or the combination of Rx1‐NES plus Rx1‐NLS trigger a hypersensitive response (HR) upon *CP106^AVR^* (Avirulent) expression, but only wild‐type (WT) Rx1 is able to prevent CP106^AVR^ protein accumulation. (a) Detection of CP105^RB^ (Resistance Breaking) and CP106^AVR^ proteins in *Rx1 Nicotiana benthamiana* plants after dexamethasone (Dex) induction, by Western blot. Ponceau S staining shows equal protein loading. (b) HR after co‐expression of *Rx1* localization variants and *Dex::CP106^AVR^* 1 day post Dex induction (1 dpda). Circles depict the infiltrated zones containing the *Agrobacterium tumefaciens* strains carrying the indicated constructs. (c) Detection of *CP105^RB^* and *CP106^AVR^* transcripts in *Rx1 N. benthamiana* plants after Dex induction, by semi‐quantitative RT‐PCR. The *EF1*α control serves as a control for equal quantities of mRNAs used in the semi‐quantitative RT‐PCR. (d) *CP105^RB^* and *CP106^AVR^* transcript levels were quantified by RT‐qPCR at 2 and 4 hpda, relative to 0 hpda, in plants co‐expressing different *Rx1* constructs. For each data point, the cycle threshold (*C*_t_) values of three replicates were normalized to the *C*_t_ values obtained for the reference genes *EF1*α and *PP2A* using the 2^−ΔΔ^ *^C^* ^t^ method. (e) Detection of coat protein (CP) by Western blot in *N. benthamiana* plants co‐expressing different *Rx1* localization variants (NLS, nuclear localization signal; NES, nuclear export signal) with either *Dex::CP105^RB^* or *Dex::CP106^AVR^*, at 4 hpda. (f) Detection of CP by Western blot in *N. benthamiana* co‐expressing different *Rx1* localization variants. CP106^AVR^ does not accumulate in the presence of Rx1 or in the presence of Rx1 variants carrying a mutant NLS (Rx1‐nls) or myristoylation motif (myr‐Rx1). CP106^AVR^ accumulates when Rx1 is sequestered in the nucleus (Rx1‐NLS) or tethered at the plasma membrane (MYR‐Rx1). For PVX‐CP CP105^RB^ or CP106^AVR^ by Western blot. For immunoblotting, proteins were extracted from *N. benthamiana* leaves at 4 hpda, unless otherwise specified. The immunoblotting was performed using polyclonal anti‐PVX antibody followed by incubation with horseradish peroxidase (HRP)‐conjugated goat anti‐rabbit immunoglobulin G (IgG) secondary antibody. The photosensitive film was exposed to the membrane 2 min before developing.

The observation that Rx1‐NES and Rx1‐NLS triggered necrosis upon PVX inoculation (Figure 1a) shows that these variants can still recognize and respond to the virus. But unlike WT Rx1 the resistance response against PVX conferred by these Rx1 variants is compromised. This conceded resistance could result from a lowered sensitivity of the immune receptor for its cognate ligand, the viral CP, and hence a delayed induction of the immune response allowing viral escape. To investigate this possibility, we expressed the CP from a dexamethasone (Dex)‐inducible promotor in the presence of the different Rx1 localization variants and monitored immune outputs such as HR development. If the mislocalized receptors exert reduced sensitivity, then a higher level of the CP will be permitted before the HR is induced. Although Rx1 confers extreme resistance to the virus without a HR, transient expression of the CP in the presence of the immune receptor results in cell death, visible as tissue necrosis (Bendahmane *et al.,* 1999). We previously generated Dex‐inducible constructs for two CP variants, one that is recognized by Rx1 (CP106^AVR^) and one that evades Rx1 recognition (CP105^RB^, for *Rx1*‐resistance breaking), and was isolated from an *Rx1* resistance‐breaking (RB) PVX strain carrying specific point mutations in the CP (Querci *et al.,* 1995). This inducible system (called CESSNA) allows us to synchronize the activation of Rx1 *in planta* (Knip *et al.,* 2019). In WT *N. benthamiana* plants that transiently express *Dex::CP105^RB^* or *Dex::CP106^AVR^*, CP105^RB^ and CP106^AVR^ proteins can be first detected at 3 and 2 h post‐Dex application (3 and 2 hdpa), respectively (Knip *et al.,* 2019). Here, we examined the levels of CP105^RB^ and CP106^AVR^ proteins in *Rx1 N. benthamiana* plants after Dex application (Figure 2a). In *Rx1*‐expressing plants transformed with the *Dex::CP106^AVR^* construct, HR becomes apparent from 4 hpda (Knip *et al.,* 2019). In line with Knip *et al* (2019), we observed that the CP105^RB^ protein was first detected at 3 hpda in the *Rx1* plants (Figure 2a). Unexpectedly, the CP106^AVR^ protein could not be detected (Figure 2a), despite the fact that an immune response was mounted (Figure 2b, *Dex::CP106^AVR^* + *Rx1*). To confirm whether the *CP* gene was expressed in the *Rx1* plants, the *CP106^AVR^* and *CP105^RB^* transcript levels were examined using semi‐quantitative RT‐PCR. Both *CP105^RB^* and *CP106^AVR^* transcripts were found to accumulate upon Dex application, and therefore our inability to detect CP106^AVR^ protein accumulation in *Rx1* plants cannot be explained by a lack of expression of the *Dex::CP106^AVR^* constructs (Figure 2c). The lower accumulation of *CP106^AVR^* transcripts, as compared with the *CP105^RB^* messenger RNA (Figure 2c), is likely to be an intrinsic property of *CP106^AVR^* as it is also less abundant in plants without *Rx1* (Figure 2d). Of note, at 4 hpda a reduction in the level of *CP106^AVR^* transcript (Figure 2c) and total protein (Ponceau S staining; Figure 2a) was observed, which is likely to be attributable to the onset of the HR at this time point, resulting in tissue collapse (Knip *et al.,* 2019).

Next, we asked whether the cytosolic or nuclear pool of Rx1 alone is sufficient and/or required to prevent CP106^AVR^ accumulation following *CP106^AVR^* expression. WT *N. benthamiana* plants were transiently co‐transformed with the following *Rx1* constructs: *Rx1*, *Rx1‐NES* and *Rx1‐NLS*, or a combination of *Rx1‐NES* and *Rx1‐NLS*, and with the *Dex::CP105^RB^* and *Dex::CP106^AVR^* constructs. Expression of the different *Rx1* constructs was confirmed by RT‐qPCR (Figure S1). Agroinfiltrated leaves were brushed with Dex and monitored for the induction of an HR. At 1 day post‐dexamethasone treatment all Rx1 variants (*Rx1*, *Rx1‐NES*, *Rx1‐NLS* and the co‐expressed *Rx1‐NES* + *Rx1‐NLS* constructs) triggered a CP106^AVR^‐specific cell death response, confirming the functionality of all constructs (Figure 2b). The accumulation of CP105^RB^ or CP106^AVR^ proteins in the presence of the various Rx1 variants was analysed at 4 hpda (Figure 2e). As expected, the non‐recognized CP105^RB^ protein accumulated both in the absence and in the presence of WT Rx1 or its NES and NLS derivatives (Figure 2e). As observed for the stable transgenic *Rx1* plants (Figure 2a), CP106^AVR^ did not accumulate when co‐expressed with WT *Rx1* (Figure 2e), but CP106^AVR^ did accumulate in the presence of Rx1‐NES or Rx1‐NLS, or in combination with Rx1‐NES + Rx1‐NLS (Figure 2d). The expression level of *CP105^RB^* or *CP106^AVR^* in the presence of Rx1‐NLS or Rx1‐NES was assessed by RT‐qPCR (Figure 2d), confirming the induction of gene expression following the application of Dex. As observed previously (Figure 2c), *CP106^AVR^* transcripts accumulated to lower levels than *CP105^RB^* transcripts in all cases (no *Rx1*, *Rx1*, *Rx1‐NLS* and *Rx1‐NES*) (Figure 2d). Nevertheless, as all genotypes show a comparable *CP106^AVR^* induction profile and expression levels, the lack of CP106 protein accumulation in the presence of Rx1 cannot be explained by a reduced expression of the *CP106^AVR^* gene.

The observation that mislocalized Rx1 variants, alone and in combination, permit CP accumulation is in support of a lower sensitivity and functionality of the mutants, allowing a higher CP accumulation before immune responses are activated. To test whether this ability is a generic property of the mislocalization of Rx1, a Rx1 variant with a myristoylation motif (MYR) was generated. In contrast to the NES variant, which can still diffuse into the nucleus, this protein will be retained in the cytoplasm, as it is anchored to the plasma membrane. When the *Dex::CP105^RB^* or *Dex::CP106^AVR^* constructs were co‐expressed with *MYR‐Rx1*, both CP variants could be detected on an immunoblot, demonstrating that mislocalized Rx1 allows CP accumulation (Figure 2f). To confirm that the loss of function does not result from the fusion of protein motifs to the Rx1 N or C termini, mutant NLS or MYR motifs were generated, resulting in Rx1‐*nls* or *myr*‐Rx1 variants. These variants restored Rx1 function, as following the application of Dex CP106^AVR^ accumulation was not detectable on an immunoblot (Figures 2f and S3). In conclusion, both WT Rx1 and its variants that are targeted to, or excluded from, the nucleus triggered cell death upon *CP106^AVR^* expression. In contrast, CP106^AVR^ accumulation was only permitted in plants co‐expressing mislocalized Rx1 variants, but not when WT Rx1 was produced. The observation that the Rx1 variants permit the CP protein to accumulate to readily detectable levels before activating the HR suggests that they are less effective in mounting an immune response as they cannot supress CP production.

### The lack of CP106^AVR^ protein accumulation in the presence of Rx1 is not caused by protein degradation

**Figure 3:**
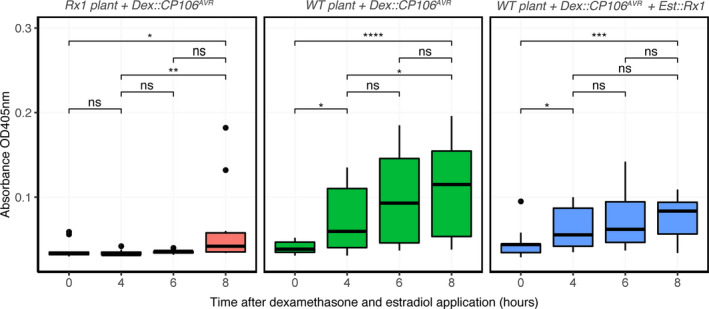
Coat protein (CP) levels remain constant following the induction of *Rx1* expression. The CP levels from *Nicotiana benthamiana Rx1* or wild‐type (WT) plants transiently transformed with *Dex::CP106^AVR^* and *Est::Rx1* were quantified by ELISA after dexamethasone (Dex) and estradiol application at *t* = 0. Statistical analysis using Wilcoxon test (ns, non‐significant; *P <0.05, **P < 0.01, ***P < 0.001, ****P < 0.0001, significant). The absorbance depicted correlates with the level of CP protein detected by ELISA.

Unlike the Rx1‐NLS, ‐NES and ‐MYR variants, the WT Rx1 conferred full viral resistance, concomitant with a strong reduction in CP106^AVR^ protein accumulation. As *CP106^AVR^* was expressed (Figure 2c,d), the reduced CP106^AVR^ protein levels could be either caused by a translational arrest of the mRNA and/or by increased turnover of the CP106^AVR^ protein upon Rx1‐mediated immune activation. To study whether, as a result of Rx1 activation, CP106^AVR^ is targeted for protein degradation by the *26S* proteasome, we assessed whether CP106^AVR^ accumulated in the presence of the proteasome inhibitor MG132 in *Rx1* plants. To this end, the *Dex::CP106^AVR^* construct was delivered using *Agrobacterium* into WT or *Rx1 N. benthamiana* leaves 24 h prior to Dex application and infiltration with MG132. Upon Dex application, the CP106^AVR^ protein levels were quantified at 0, 1, 2, 3 and 4 hpda in leaves with or without MG132 treatment (Figure S4). Irrespective of the treatment with the proteasome inhibitor (MG132), CP106^AVR^ could not be detected in the presence of Rx1 (Figure S4). To further exclude the possibility of degradation of CP106^AVR^ by other mechanisms, we quantified the CP106^AVR^ protein levels and turnover using a set‐up in which CP106^AVR^ was allowed to accumulate prior to *Rx1* expression. A swift decrease in the CP106^AVR^ protein levels as a result of Rx1 activation would be indicative of (targeted) protein degradation, whereas a translational arrest is predicted to result in a fairly constant level of CP106^AVR^ following Rx1 activation. An estradiol‐inducible *Rx1* construct (*Est::Rx1*) was developed to control *Rx1* expression independently from that of *CP106^AVR^*. The optimal time points for the application of the inducers (Dex and estradiol) for a reliable quantification of CP106^AVR^ levels upon *Rx1* induction were determined empirically. Thereto WT *N. benthamiana* plants were transiently transformed with *Est::Rx1* and *Dex::CP106^AVR^* constructs. The inducers were added at different time points and the accumulation of CP106^AVR^ was determined by ELISA. We observed that the simultaneous induction of *CP106^AVR^* and *Rx1* expression by mixing the two inducers allowed the accumulation of CP106^AVR^ to detectable levels before observing a Rx1 response. A possible explanation for this finding is that CP106^AVR^ is permitted to accumulate until a signalling‐competent Rx1 protein is formed. As controls for this set‐up, CP106^AVR^ accumulation was quantified in the absence of Rx1 (e.g. in WT plants) and in stable transgenic *Rx1* plants. In *Rx1* plants (constitutively expressing *Rx1*) agroinfiltrated with the *Dex::CP106^AVR^* construct, only trace levels of CP were detected upon Dex/estradiol application (Figure 3, left panel), confirming our earlier observation that CP106^AVR^ accumulation is compromised in the presence of Rx1 (Figure 2a,c). As expected, in WT plants agroinfiltrated with the *Dex::CP106^AVR^* constructs, the level of CP106^AVR^ increased over time upon Dex application (Figure 3, middle panel). When *Rx1* and *CP106^AVR^* expression were induced simultaneously by co‐applying Dex and estradiol, the level of CP106^AVR^ protein increased from 0 to 4 hpda, but then remained constant until the last recorded time point at 8 hpda (Figure 3, right panel). As no apparent decrease of CP106^AVR^ was observed over this time period this implies that CP106^AVR^ was not specifically degraded after Rx1 activation. All together, these results suggest that the lack of CP106^AVR^ accumulation upon Rx1 activation is not linked with increased protein turnover, or protein degradation, but is possibly linked with translational arrest of the *CP106^AVR^* transcript.

### Rx1 immune activation does not induce deadenylation or decapping of CP transcripts

**Figure 4:**
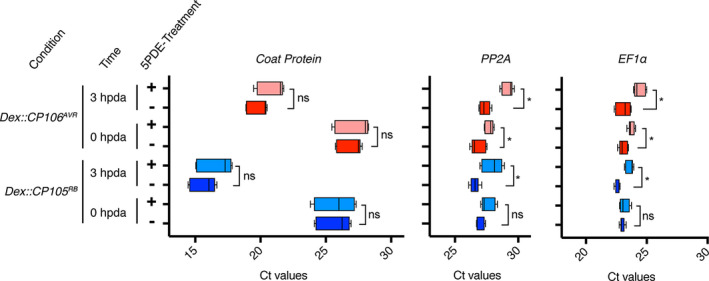
Coat protein (CP) transcripts and endogenous transcripts are not massively deadenylated or decapped after Rx1 activation. Transcript quantification by RT‐qPCR on cDNA generated with oligo‐dT from total RNA (untreated) or capped RNA only (5PDE treated) from *Rx1* plants agroinfiltrated with *Dex::CP105^RB^* or *Dex::CP106^AVR^* constructs at 0 and 3 hpda. Cycle threshold (*C*_t_) values from three biological replicates and three technical replicates are plotted. Statistical analysis using Kruskal–Wallis test (ns, non‐significant; *P < 0.01, significant).

Eukaryotic mRNAs need a 5′ cap and a 3′ poly‐A tail to be translated by the ribosomes into a polypeptide. Because the *CP* transcripts were readily detected using semi‐quantitative RT‐PCRs (Figure 2c), it can be deduced that the *CP106^AVR^* mRNAs are polyadenylated, as the cDNA was generated with oligo‐dT primers that anneal to the poly‐A tail. To check whether the *CP* transcripts have a protective 5′ cap, we used a 5′‐phosphate‐dependent exonuclease (5PDE) that degrades RNAs that lack a 5′ cap. Total RNA was isolated at 0 and 3 hpda from *Rx1* plants transiently expressing *Dex::CP105^RB^* or *Dex::CP106^AVR^* constructs. Each total RNA sample was split into two: one sample was treated with 5PDE to degrade any uncapped RNA, whereas the other sample remained untreated. Agarose gel electrophoreses of treated and non‐treated samples confirmed the depletion of ribosomal RNAs after 5PDE treatment, showing the effectiveness of the exonuclease to degrade uncapped RNAs (Figure S5). cDNAs were subsequently generated from untreated and 5PDE‐treated samples using oligo‐dT primers to reverse transcribe any remaining mRNAs (i.e. RNA with a 3′ poly‐A tail). Quantitative real‐time PCRs (RT‐qPCRs) were performed on these cDNA samples, from both 5PDE‐treated and non‐treated RNAs, to determine whether the mRNAs remain capped and polyadenylated after Rx1 immune activation (Figure 4). First, to assess whether 5PDE treatment did not affect general transcript integrity we examined the mature mRNA levels of two endogenous reference genes (*PP2A* and *EF1α*). These genes were selected as their transcript levels were reported to remain fairly constant upon Rx1 activation (Knip *et al.,* 2019). RT‐qPCR analysis confirmed that both transcripts are constitutively expressed following Rx1‐induced immune signalling (Figure 4, compare the dark‐red and dark‐blue cycle threshold, *C*_t_, values for *PP2A* and *EF1α* transcripts). Notably, a small but significant difference in *C*_t_ values could be observed between treated and untreated samples for these reference genes, indicating that a small portion of the *PP2A* and *EF1α* mRNAs are uncapped in the *Dex::CP106^AVR^* samples at 0 and 3 hpda and in the *Dex::CP105^RB^* samples at 3 hpda. (Figure 4). As similar differences were detected in *Dex::CP105^RB^* and *Dex::CP106^AVR^* samples, this observation implies that the decapping of *PP2A* and *EF1α* transcripts is not related to Rx1‐mediated immune activation, but is likely to reflect their natural turnover *in planta*. Regarding the *CP* transcripts, as expected, a clear induction of their expression could be observed upon Dex treatment (Figure 4, diminution of *C*_t_ values for the *CP* between 0 and 3 hpda). Notably, when comparing the *C*_t_ values between the 5PDE‐treated (capped) and non‐treated (total) samples, no significant differences were observed (Figure 4, left panel). The fact that these *C*_t_ values did not differ, implies that the majority of the *CP105^RB^* and *CP106^AVR^* transcripts at the different time points post Dex treatment are both 5′ capped and 3′ poly‐A tailed. If they were to be decapped, a difference between the 5PDE‐treated and non‐treated sample was expected. Together, these results show that *CP* transcripts remain capped and poly‐A tailed following Rx1 activation, implying that arrested translation is a likely cause for the lack of CP accumulation.

### Rx1 does not trigger a general translational arrest, as *de novo* protein synthesis occurs upon immune activation

**Figure 5:**
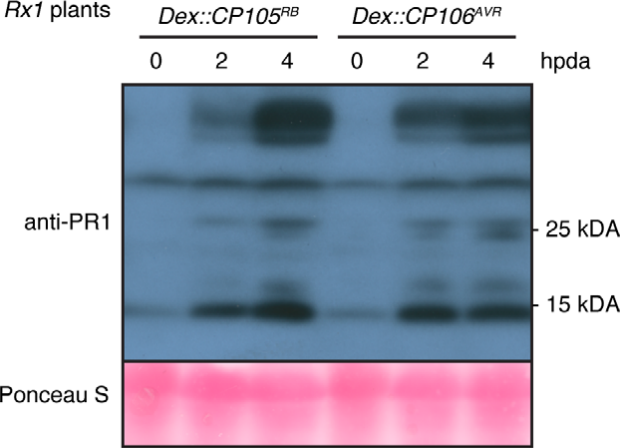
PR1 proteins accumulate after dexamethasone (Dex) induction of coat protein (CP) expression in *Rx1* plants. Immunodetection of PR1 proteins in *Rx1* plants agroinfiltrated with *Dex::CP105^RB^* or *Dex::CP106^AVR^*, at 0, 2 and 4 hpda.

Rx1 immune activation prevents the accumulation of CP106^AVR^, even though the *CP106^AVR^* transgene is transcribed and the resulting mature mRNA appears to be intact. To assess whether Rx1 activation triggers a global translational arrest of *de novo* produced transcripts, we monitored whether the synthesis of proteins other than CP106^AVR^ occurred upon Rx1 activation. As the transcript levels of *pathogenesis‐related 1* (*PR1*) are induced after Rx1 activation, we determined PR1 protein accumulation (Knip *et al.,* 2019). Thereto *Rx1 N. benthamiana* was transiently transformed with *Dex::CP105^RB^* or *Dex::CP106^AVR^* constructs and the leaves where brushed with Dex 2 days after agroinfiltration. At 0, 2 and 4 hpda, the total protein fraction was isolated from the treated leaves and used for immunoblotting. Probing the blots with a polyclonal antibody raised against PR1 (using the N‐terminal part of a recombinant PR1 protein from *Arabidopsis thaliana*) revealed that the PR1 protein levels increased in response to treatment with Dex (Figure 5). The accumulation of this *Nb*PR1 homologue after Rx1 immune activation thus indicates that Rx1 activation does not cause global translational arrest.

### Rx1‐mediated CP translational arrest is not inhibited by the viral silencing suppressors p19 and p38

**Figure 6:**
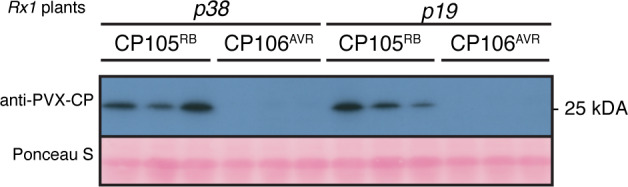
The viral suppressor of silencing (VSR) p38 does not inhibit the Rx1‐mediated translational arrest of CP106^AVR^. Coat protein (CP) accumulation in three independent *Rx1* *Nicotiana benthamiana* plants transiently expressing *Dex::CP105^RB^* or *Dex::CP106^AVR^* in combination with the VSRs p38 and p19, as detected by Western blot using PVX‐CP antibodies. Proteins were extracted at 4 hpda.

Upon elicitation of the tobacco N protein by the viral p50 avirulence protein of TMV, a translational arrest can be triggered in *N. benthamiana* that also targets specific PVX transcripts (Bhattacharjee *et al.,* 2009). The N‐mediated translational arrest of *PVX* transcripts is inhibited by the silencing suppressor p38 from turnip crinkle virus (TCV) (Thomas *et al.,* 2003), but not by the p19 silencing suppressor from a *Tombusvirus*, such as the cymbidium ringspot virus (CymRSV) (Silhavy *et al.,* 2002). To determine whether the Rx1‐mediated translational arrest of *PVX‐CP* transcripts is similarly inhibited by these silencing suppressors, p38 or p19 were co‐expressed with the *Dex::CP105^RB^* or *Dex::CP106^AVR^* constructs in *Rx1 N. benthamiana* using agroinfiltration. The functionality of both silencing suppressors was confirmed by co‐expressing p38 or p19 with *GFP* and both were found to boost GFP fluorescence intensity (Figure S6). As for the tobacco *N* gene, apparently the expression of p19 did not block the Rx1‐mediated translational arrest of *CP106^AVR^*, as the CP106^AVR^ protein remained undetected in *Rx1* plants (Figure 6). Unlike N‐mediated translational arrest, p38 did not inhibit the Rx1‐mediated translational arrest of *CP106^AVR^*, as no CP106^AVR^ protein was detected in *Rx1* plants in the presence of p38. Combined, our data suggest that Rx1 mounts a translational arrest of the CP transcript using a pathway distinct from the N‐mediated translational arrest, as p38 interfered with N but not with Rx1 function.

## DISCUSSION

In this study we show that although Rx1 triggers extreme resistance upon PVX rub‐inoculation, Rx1 localization variants that are either nuclear localized or nuclear excluded (Rx1‐NLS and Rx1‐NES) fail to trigger viral resistance in *N. benthamiana*. Nevertheless, Rx1‐NES and Rx1‐NLS still induce cell death upon PVX inoculation, showing that these Rx1 variants are capable of recognizing and responding to the virus with an HR. The capacity of Rx1 to trigger extreme resistance coincides with the ability of Rx1 to interfere with PVX‐CP protein accumulation (and potentially other PVX proteins). Our data strongly suggest that this is linked with the translational arrest of CP mRNAs and is not the result of global translational arrest.

Our results show that cell death is neither sufficient nor required for Rx1‐mediated PVX resistance, but that a translational arrest of the *CP106^AVR^* transcript correlates with full immunity. These observations argue that extreme resistance conferred by Rx1 is somehow linked to a swift translational arrest of viral transcripts, preventing the invading virus from replicating and spreading. Apparently, the level of CP present on a viral particle is sufficient to trigger ER and halt the virus, preventing it to transcribe its genome and produce CP. When the CP is produced heterologously, for instance by agroinfiltration or using inducible promotors, CP transcripts are allowed to accumulate prior to CP protein presence, enabling the production of the protein before its translation can be halted. Furthermore, these findings imply that cell death is induced by Rx1 when CP protein levels exceeds a threshold concentration. Hence, when Rx1 variants are used that cannot block CP translation the protein produced will exceed this threshold, resulting in cell death (Figure 2b) or trailing necrosis following viral infection (Figure S2). Interestingly, when *Rx1‐NES* and *Rx1‐NLS* were co‐expressed they also failed to trigger the translational arrest of CP (Figure 2d). This observation shows that the mere presence of Rx1 in the nucleus and cytosol is not sufficient to block CP translation, implying that the R protein needs to be able to shuttle between the cytosol and the nucleus.

Translational arrest as an antiviral immune response has been reported in mammalian and plant systems (Machado *et al.,* 2017). In humans, infection by Rift Valley fever virus (RVFV) is followed by a translational shutdown that restricts viral infection (Hopkins *et al.,* 2015). In Arabidopsis, the activation of the leucine‐rich repeat receptor‐like kinase (LRR‐RLK) NIK1, triggered by begomovirus infection, leads to global translational arrest (Zorzatto *et al.,* 2015). This translational arrest, which includes viral transcripts, contributes to an enhanced resistance to the virus. Rx1‐mediated translational arrest appears to be distinct, as the *de novo* protein synthesis, such as PR1s, still occurred after immune activation. These different outputs imply that different signalling routes are involved.

Specific responses targeted towards viral transcripts have been described during viral recovery. Viral recovery has been reported in several plant–virus interactions and is hallmarked by a decrease of disease symptoms and viral titres in developing leaves following systemic viral infection. Translational repression of viral transcripts is probably involved in this phenomenon. In fact, it has been shown that viral RNAs of TRV in recovered plants associate less with ribosomes and accumulate in processing bodies (PBs), which are cytoplasmic foci where translationally repressed mRNAs are stored and eventually processed by decapping enzymes and exoribonucleases (Ma *et al.,* 2014). Interestingly, the TNL N from tobacco, which confers resistance to TMV, can trigger a translational arrest of PVX transcripts in *N. benthamiana* (Bhattacharjee *et al.,* 2009; Meteignier *et al.,* 2016). Immune activation of N, following recognition of the p50 fragment of the TMV replicase, prevents *PVX* transcripts associating with the ribosomal proteins required for their translation. This repression is specific for viral transcripts and sequences inserted in the viral genome and does not involve global translational arrest and is associated with the formation of PBs (Meteignier *et al.,* 2016). Hence, both a TNL (N) and a CNL (Rx1) are capable of triggering a translational arrest of viral transcripts after immune activation. Concerning N, different components of the RNA silencing machinery appear to be involved as for Rx1. The VSRs p38 from TCV (which interferes with the loading of double‐stranded RNA into the AGO1/2‐RISC complex; Iki *et al.,* 2017) is able to interfere with the induction of the translational arrest mediated by N (Bhattacharjee *et al.,* 2009). Interestingly, the Rx1‐mediated translational arrest of *PVX‐CP* is not inhibited by this VSR, suggesting that N and Rx1 trigger the translational arrest of *PVX‐CP* mRNAs via distinct mechanisms. Furthermore, N*‐*mediated translational arrest is accompanied by the formation of PBs, in which translationally inhibited transcripts are most likely decapped (Meteignier *et al.,* 2016). Our results show that during Rx1 immunity, CP transcripts are not massively decapped, again suggesting diversity in the translational arrest mechanism triggered by N and Rx1.

The observation that two unrelated NLRs, the CNL Rx1 and the TNL N, both activate translational inhibition implies that it could be a common defence output of NLRs. Whereas translational inhibition conceptionally poses a relevant defence strategy against viral pathogens, its usefulness against other types of pathogens is less obvious. Interestingly, important changes in the translatome (transcripts associated with polyribosomes for translation) are observed within 2 h of immune activation of RPM1, which is an NLR conferring resistance against a bacterium (Meteignier *et al.,* 2017). Specifically, the authors reported a diminution in the association between *target of rapamycin* (*TOR*) transcripts and polyribosomes during RPM1 immunity. TOR is a conserved and important factor in translation signalling, often hijacked and hyperactivated by both plant and mammalian viruses to enhance the translation of their proteins (Ouibrahim *et al.,* 2015, Le Sage *et al.,* 2016). This finding suggests a tight link between NLR activation and translational regulation. Determining the contribution of this response to immunity requires exploring the capacity of other NLR proteins to interfere with the translation of (viral) transcripts in future studies. The tools described in this study enable the dissection of molecular mechanisms underlying specificity towards viral (or non‐self) transcripts by the NLR Rx1. Importantly, this study opens new perspectives in the mechanisms underlying NLR‐mediated extreme resistance against viruses.

## Experimental procedures

### Plasmids

The Dex‐inducible CP constructs, *Dex::CP105^RB^* and *Dex::CP106^AVR^*, are described by Knip *et al.* (2019). To generate Rx1 localization variants, the *Rx1* open reading frame (ORF) was cloned into pBINPLUS vectors, with a leaky scan (35S_LS_) promoter, different cellular localization signal (myristoylation = MYR, nuclear localization signal = SV40 NLS and nuclear export signal = PKI NES, or a mutant version of these signal peptides) and a C‐terminal GFP or Flag tag. The estradiol‐inducible Rx1 construct, referred to as *EST::Rx1*, was generated by inserting the *Rx1* ORF into the pER10 vector (Zuo *et al.,* 2000), after an estradiol‐inducible promoter, into the *SgsI* restriction site. The pBIN61 constructs to express the viral suppressor of silencing p19 and p38 were previously described (Ma *et al.,* 2015; Thomas *et al.,* 2003).

### Plant lines and *Agrobacterium*‐mediated transformation of N. benthamiana

Stable transgenic *N. benthamiana* producing Rx1 variants were generated as described in Richard *et al.* (2020), using the *35S_LS_::Rx1‐NES‐GFP* and *35S_LS_::Rx1‐NLS‐GFP* constructs described previously. Briefly, *N. benthamiana* plants were transformed using *Agrobacterium*‐mediated transformation as described by Sparkes *et al.* (2006). *Agrobacterium tumefaciens* GV3101‐infiltrated leaves were surface sterilized, cut into 2‐cm^2^ diamond shaped pieces and placed on shoot‐induction medium supplemented with 50 μg ml^−1^ kanamycin and 2.5 μg ml^−1^ benomyl for selection. Shoots from putative transformants were transferred to root‐induction medium containing 50 μg ml^−1^ kanamycin. Sixteen candidate transformants for both *35S_LS_::Rx1‐NES‐GFP* and *35S_LS_::Rx1‐NLS‐GFP* constructs were selected for seed production. Segregation for kanamycin resistance of the obtained T_1_ progeny was assessed on selective medium and five and three lines with a single insertion were identified for *35S_LS_::Rx1‐NES‐GFP* and *35S_LS_::Rx1‐NLS‐GFP* constructs, respectively. The homozygosity of the T_2_ generation was evaluated by RT‐qPCR using genomic DNA through an estimation of the t‐DNA copy number (by amplification of the kanamycin resistance gene, using the oligonucleotides FP7724‐NPTII‐FW, 5′‐TCACCTTGCTCCTGCCGAGA‐3′, and FP7725‐NPTII‐RV, 5′‐CGAGCCCCTGATGCTCTTCG‐3′), compared with an endogenous reference gene (*NRG1*, amplified using the oligonucleotides FP8254, 5′‐GTGTCCGACCACTAAGCATGGAACTA‐3′, and FP8255, 5′‐CTGCTGGTGCATCCTTTCTGGAAATC‐3′), as described by Richard *et al.* (2020). The homozygous line *35S_LS_::Rx1‐NES‐GFP #6‐2* and *35S_LS_::Rx1‐NES‐GFP #6‐7* were selected. The *35S_LS_::Rx1‐NES‐GFP #6‐2* line presented a phenotype similar to WT plants, whereas *35S_LS_::Rx1‐NLS‐GFP #6‐7*, the only homozygous line that produced seeds, presented a dwarfed and slightly bleached phenotype.

Both WT and transgenic *Rx1:4xHA*, *35S_LS_::Rx1‐NES‐GFP* and *35S_LS_::Rx1‐NLS‐GFP N. benthamiana* were grown under long‐day conditions in a climate chamber for 4–5 weeks (22°C, 70% relative humidity, 11‐h light/13‐h dark cycle). *Agrobacterium*‐mediated transformation was performed on the youngest fully expanded leaves (Ma *et al.,* 2012). *Rx1* and *Dex::CP* constructs were infiltrated at an OD_600_ of 0.05 and 0.2, respectively. The silencing suppressor constructs were infiltrated at an OD_600_ of 0.5. Dex induction was performed in the morning, 2 days following agroinfiltration, by brushing 20 μm Dex, 0.01% Silwet L‐77, in ultrapure water on the surface of the leaves.

The accumulation and subcellular localization of the different Rx1‐GFP proteins in *N. benthamiana* was assessed by confocal microscopy. Imaging was performed on an LSM510 (Zeiss, https://www.zeiss.com), GFP was excited at 488 nm with an Ar‐ion laser and emission was recorded at 505–530/550 nm.

### PVX rub‐inoculation

To produce infectious PVX‐GFP particles, leaves of 4 week‐old WT *N. benthamiana* plants were agroinfiltrated with an *A. tumefaciens* GV3101 strain containing the pJIC SA_Rep helper plasmid and the *PVX‐GFP* construct, as previously described (internal identifier BglFP#4081; Richard *et al.,* 2020). Two weeks after agroinfiltration, systemically infected leaves were either snap frozen with liquid nitrogen and stored at −80°C or directly used for rub‐inoculation. Four week‐old *N. benthamiana* plants were rub‐inoculated with PVX‐GFP inoculum, as described by Richard *et al.* (2020). Disease symptom development was followed over time and pictures were taken using a Lumix DMC‐LX15 camera (Panasonic, https://www.panasonic.com) under normal light or placed in a dark chamber to detect viral spread (Extraneous Light Protector and RS 1 stand; Kaiser Fototechnik, https://kaiserfotous.com), illuminated with UV light (RB 5003 UV Lighting Unit code no. 5591; Kaiser Fototechnik).

### Protein isolation, Western blot, dot blot and antibodies

For PVX, PVX‐CP and PR1 detection by immunoblot, proteins were isolated and immunodetection was performed as described by Knip *et al.* (2019) using polyclonal antibody raised against PVX (diluted 1:3000) (ref. 110411; Bioreba, https://www.bioreba.ch), followed by incubation with horseradish peroxidase (HRP)‐conjugated goat anti‐rabbit immunoglobulin G (IgG) secondary antibody (diluted 1:10 000) (ref. 31460; ThermoFisher Scientific, https://www.thermofisher.com) for PVX or PVX‐CP detection using a homemade ECL solution: Tris‐HCL, pH 8.5, 0.1 m, coumaric acid 0.2 mm (Sigma‐Aldrich, https://www.sigmaaldrich.com) and luminol 1.25 mm (Fluxa, https://www.fluxa.io). PR1 proteins were detected using PR1 polyclonal antibody (diluted 1:2500) (ref. AS10 687; Agrisera, https://www.agrisera.com) as the primary antibody and the same HRP‐conjugated secondary antibody as mentioned above.

For the *26S* proteasome inhibition experiment, 2 days after agroinfiltration, agroinfiltrated leaves were infiltrated with 100 μm MG132 (ref. 3175‐v; Peptide International, now Vivitide, https://vivitide.com) in 2‐(*N*‐morpholine)‐ethanesulphonic acid (MES), pH 5.6, plus 1% DMSO, 3 h before Dex induction. Proteins were sampled at 4 h post Dex application and isolated as described above. The dot blot was performed by spotting total protein extracts on an activated polyvinylidene difluoride (PVDF) membrane lying on top of two wet (Tris‐buffered saline, TBS) and five dry Whatman papers (from top to bottom). After protein spotting, the membranes were checked with Ponceau S staining for the presence of proteins. The immunodetection was performed as described above for the immunobloting method, using the same polyclonal antibody raised against PVX (diluted 1:3000).

### Dexamethasone and estradiol induction on plate and CP quantification by ELISA

Two days post‐agroinfiltration of *N. benthamiana* WT and *Rx1* with *A. tumefaciens* GV3101 carrying *Dex::CP106^AVR^* (infiltration OD_600_ 0.1) and/or *Est::Rx1* (OD_600_ 0.1) constructs, eight leaf discs of 6 mm in diameter were sampled and placed in 1 ml of Dex 20 μm, estradiol 10 μm and 0.01% Silwet L‐77, in ultrapure water. At the indicated time points, leaf discs were collected, dried quickly on Whatman paper and flash frozen in liquid nitrogen. Leaf material was homogenized in 50 mm sodium phosphate buffer, pH 7, using a TissueLyser II grinding mill (Qiagen, https://www.qiagen.com) with three 3‐mm steel balls at 30 Hz for 30 sec, twice. The PVX‐CP protein levels were determined using a double antibody sandwich (DAS) ELISA with PVX antibodies (Prime Diagnostics, https://www.wur.nl/nl/show/Prime‐Diagnostics.htm), as described by Richard *et al.* (2020).

### RNA isolation, RT‐PCR and RT‐qPCR and terminator treatment

Total RNA was extracted from ground plant tissues using TRIzol LS reagent according to the supplier’s protocol (ThermoFisher Scientific). The RNA obtained was treated with DNase (ThermoFisher Scientific) according to the supplier’s protocol. RNA concentrations were determined by measuring the absorbance at 260 nm, Abs(260), on a Nanodrop (ThermoFisher Scientific). cDNA was synthesized from 1 μg of total RNA using RevertAid H reverse transcriptase and oligo‐dT (Eurofins, https://www.eurofins.com) in the presence of the RNAse inhibitor Ribolock (ThermoFisher Scientific), following the supplier’s protocol, and diluted 10 times in RNAase‐free double‐distilled water.

The semi‐quantitative RT‐PCR (25 cycles, annealing temperature of 60°C) was performed on 1 μl of diluted cDNA using DreamTaq DNA Polymerase (ThermoFisher Scientific) following the supplier’s protocol, using CP‐specific primers FP8371‐PVX‐CP‐F, 5′‐CACTGCAGGCGCAACTCC‐3′, and FP8372‐PVX‐CP‐R, 5′‐GTCGTTGGATTGYGCCCT‐3′, or *EF1α* primers FP8391‐NbEF1α‐F, 5′‐AGCTTTACCTCCCAAGTCATC‐3′, and FP8392‐NbEF1α‐R, 5′‐AGAACGCCTGTCAATCTTGG‐3′, as a positive internal control.

The RT‐qPCRs were performed in a QuantStudioTM3 (ThermoFisher Scientific), using the 5 × HOT FirePolEvaGreen qPCR Mix Plus, with passive reference dye ROX (Solis BioDyne, https://www.solisbiodyne.com). The 10 μl of PCR mix contained 10 pm of each primer and 2 μl of 10× diluted cDNA. The cycling programme was set to 15 min at 95°C, 40 cycles of 15 sec at 95°C, 20 sec at 60°C and 30 sec at 72°C, followed by a melting curve analysis of 15 sec at 95°C, 1 min at 60°C and 15 sec at 95°C. PVX‐CP, EF1α, protein phosphatase 2A (PP2A) and Rx1 were amplified using FP8371‐PVX‐CP‐F, 5′‐CACTGCAGGCGCAACTCC‐3′, FP8372‐PVX‐CP‐R, 5′‐GTCGTTGGATTGYGCCCT‐3′, FP8391‐NbEF1α‐F, 5′‐AGCTTTACCTCCCAAGTCATC‐3′, FP8392‐NbEF1α‐R, 5′‐AGAACGCCTGTCAATCTTGG‐3′, and FP8369‐NbPP2A‐F, 5′‐GACCCTGATGTTGATGTTCGCT‐3′, FP8370‐NbPP2A‐R, 5′‐GAGGGATTTGAAGAGAGATTTC‐3′, FP6990‐Rx1‐F, 5′‐AGCATCTGAAAGGCAGGAGA‐3′, FP8704‐Rx1‐R, 5′‐ATTCCAACTTTCGTCAAAATTC, respectively.

To evaluate the presence of 5′ Cap on mRNA, total RNA samples were isolated as described above and treated with the Terminator^™^ 5′‐phosphate‐dependent exonuclease (Lucigen, https://www.lucigen.com): two 1‐μg aliquots of total RNA each were incubated for 1 h at 30°C with 1× Terminator buffer A, 0.5 μl of Ribolock (ThermoFischer Scientific) and 1 μl of Terminator exonuclease for the treated samples (‘capped’), or 1 μl of RNAase‐free double‐distilled water for the mock‐treated samples (‘total’). Directly after the nuclease treatment, the RNA was cleaned with the RNAeasy mini kit (Qiagen) following the supplier’s protocol for RNA clean‐up and eluted in 20 μl of RNAase‐free double‐distilled water. The efficiency of the Terminator exonuclease treatment was evaluated on agarose gel. The resulting 15 μl of RNA was treated with DNAse and reverse transcribed into cDNA as described above using oligo‐dT_19_ primer. The RT‐qPCRs were performed as described above on 1 µl of 10× diluted cDNA.

## Author Contributions

MMSR and FLWT designed the study and wrote the article. MMSR, MK, JS and MSB performed the experiments. MMSR analysed the data and drafted all the figures. All authors read and approved the final version for publication.

## Conflict of Interest

The authors have no conflicts of interest to declare.

## Supporting information


**Figure S1**. RT‐qPCR analysis to monitor *Rx1* expression in *Nicotiana benthamiana*.

**Figure S2.** Rx1‐NLS and Rx1‐NES variants cannot restrict PVX‐GFP replication and spread, resulting in trailing necrosis.

**Figure S3.** The hypersensitive response (HR) after co‐expression of *myr*‐*Rx1* and *MYR‐Rx1* in the presence of *Dex::CP106^AVR^* at 1 day post Dex application (1 dpda), indicated by red circles; absence of HR in the presence of *Dex::CP105^RB^*, indicated by blue circles.

**Figure S4**. Treatment with the proteasome inhibitor MG132 does not restore CP106^AVR^ protein accumulation in the presence of Rx1.

**Figure S5**. Verification of the 5′‐phosphate‐dependent exonuclease (5PDE) treatment.

**Figure S6**. Confirmation of the silencing suppression activity of p38 and p19 on *35S::GFP* transgene expression in *Rx1 Nicotiana benthamiana* plants.

## Data Availability

All relevant data can be found within the article and its supporting materials.
