# Supplementary figures and images for: Perturbation of nuclear–cytosolic shuttling of Rx1 compromises extreme resistance and translational arrest of potato virus X transcripts
Source: Plant J. 2021 Mar 23;106(2):468–79. doi: 10.1111/tpj.15179 (PMC8252585; doi:10.1111/tpj.15179)

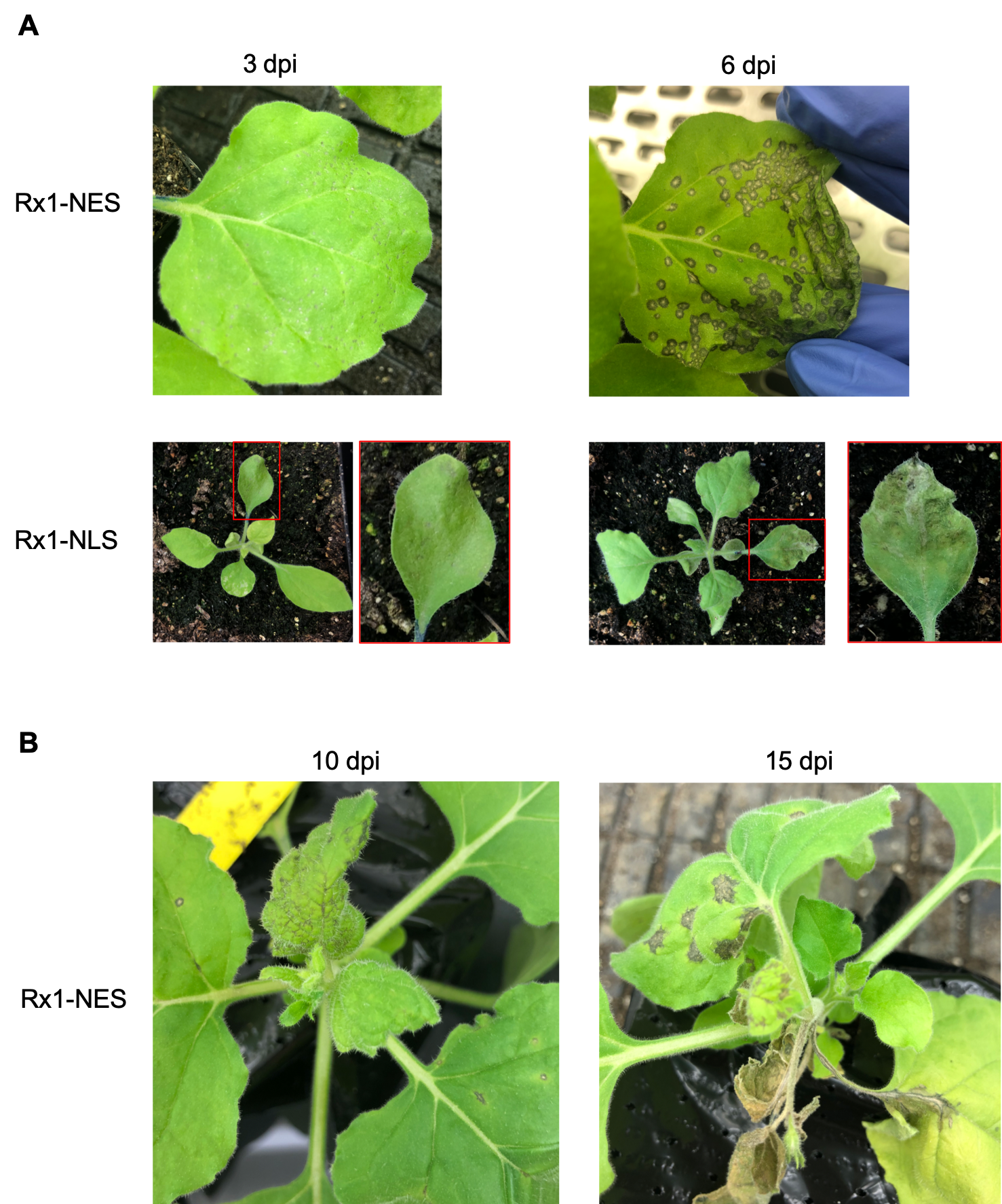

Supplement: Supplementary file 2 — Figure S2. Rx1‐NLS and Rx1‐NES variants cannot restrict PVX‐GFP replication and spread, resulting in trailing necrosis. [file TPJ-106-468-s006.png]

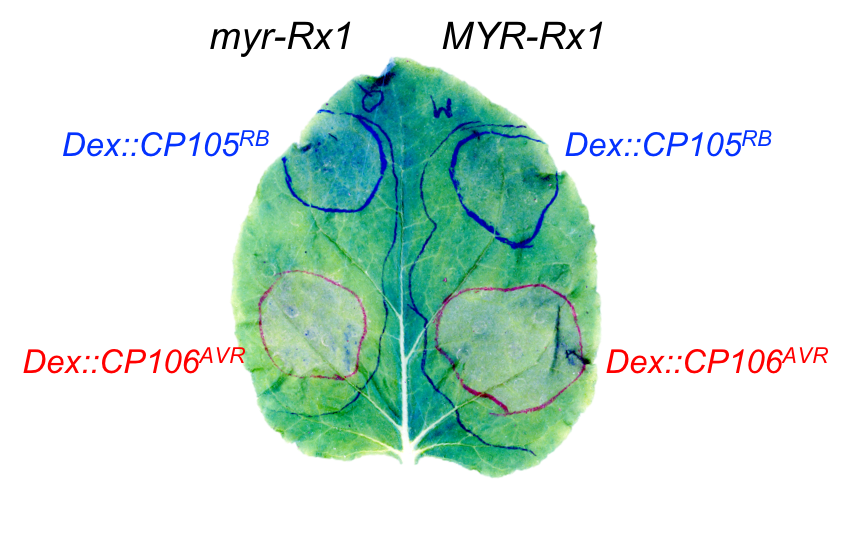

Supplement: Supplementary file 3 — Figure S3. The hypersensitive response (HR) after co‐expression of myr‐Rx1 and MYR‐Rx1 in the presence of Dex::CP106AVR at 1 day post Dex application (1 dpda), indicated by red circles; absence of HR in the presence of Dex::CP105RB, indicated by blue circles. [file TPJ-106-468-s002.png]

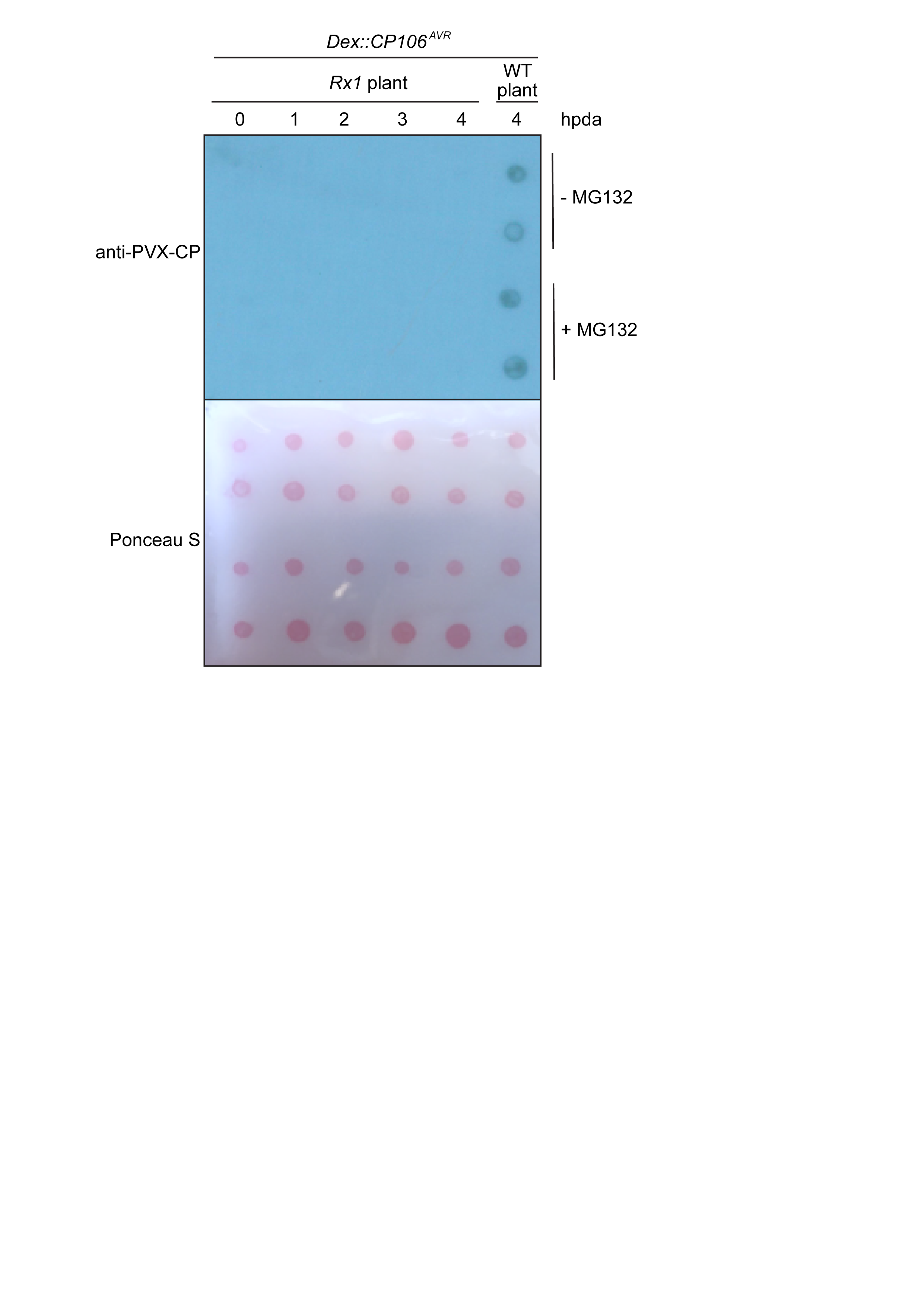

Supplement: Supplementary file 4 — Figure S4. Treatment with the proteasome inhibitor MG132 does not restore CP106AVR protein accumulation in the presence of Rx1. [file TPJ-106-468-s004.tif]

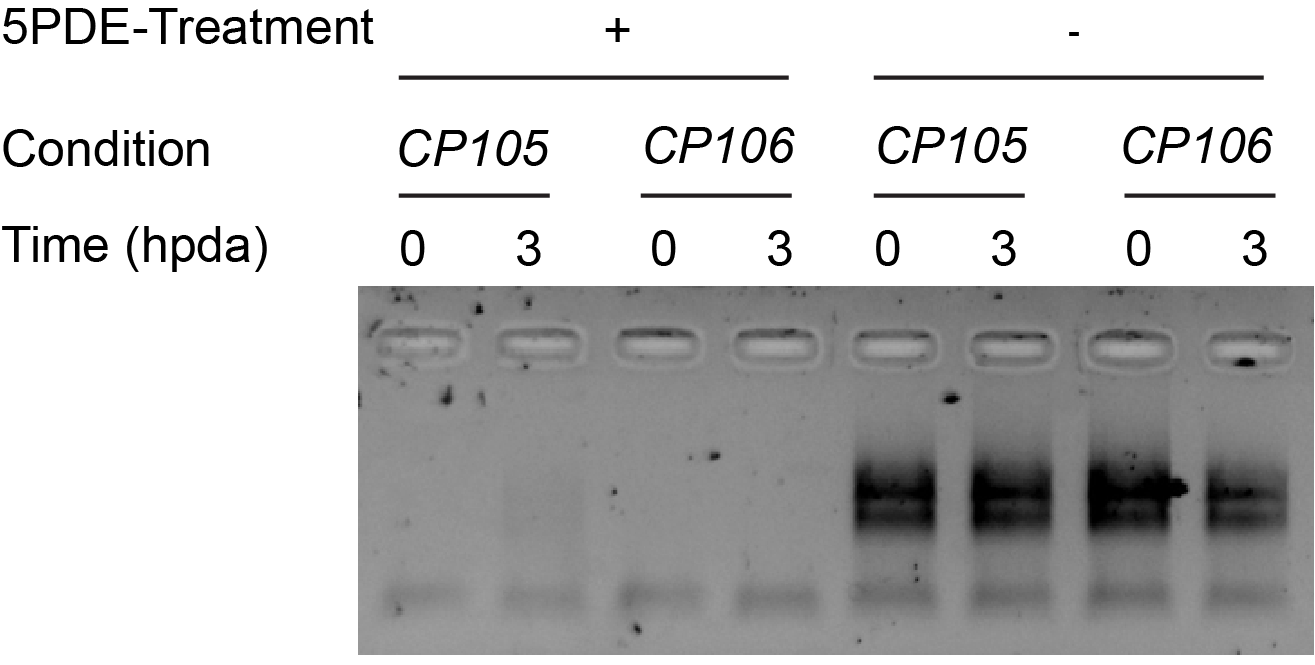

Supplement: Supplementary file 5 — Figure S5. Verification of the 5′‐phosphate‐dependent exonuclease (5PDE) treatment. [file TPJ-106-468-s001.png]

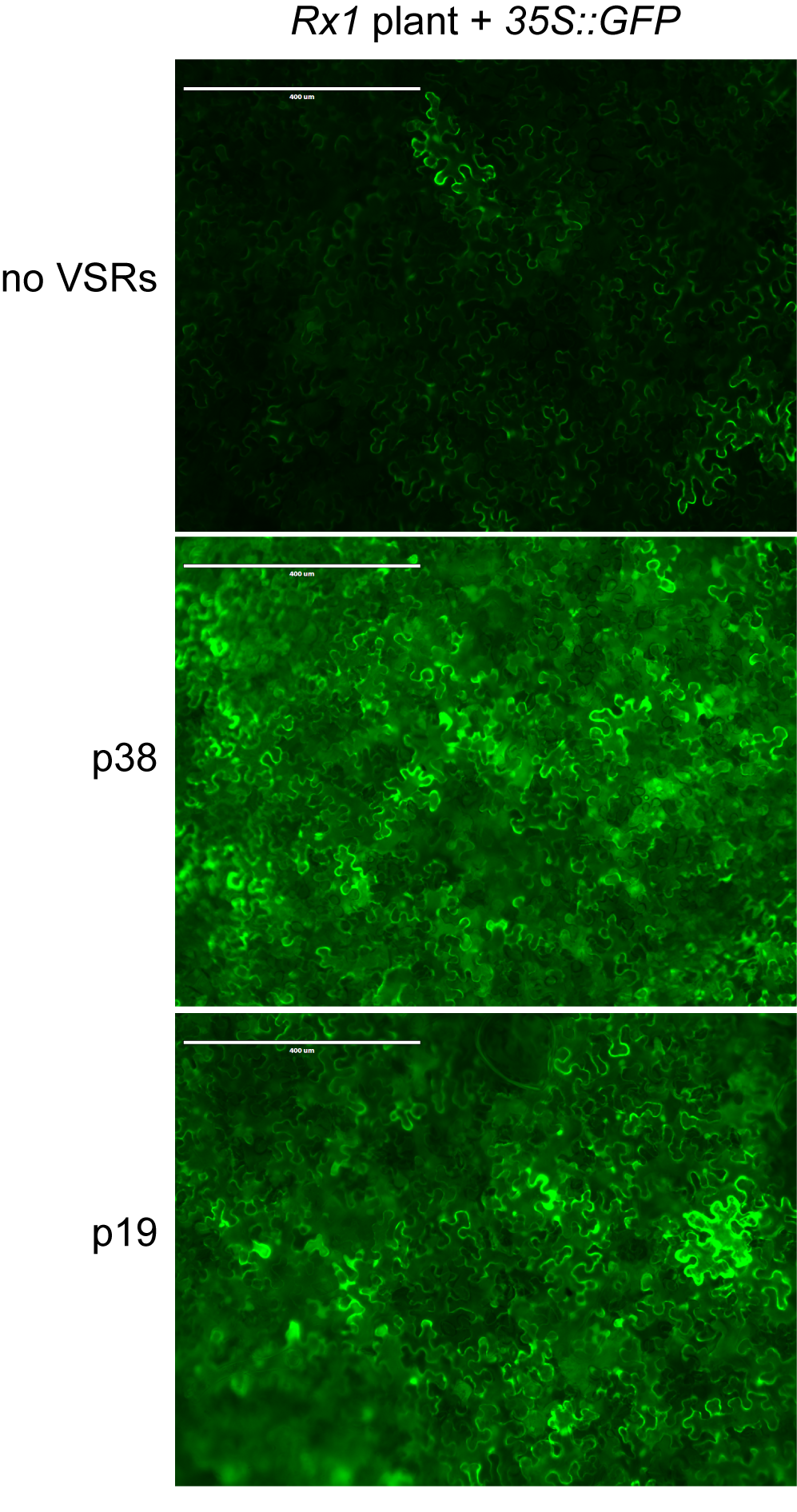

Supplement: Supplementary file 6 — Figure S6. Confirmation of the silencing suppression activity of p38 and p19 on 35S::GFP transgene expression in Rx1 Nicotiana benthamiana plants. [file TPJ-106-468-s003.tif]
